# Overnight Melatonin Concentration and Sleep Quality Are Associated with the Clinical Features of Polycystic Ovary Syndrome

**DOI:** 10.3390/biomedicines11102763

**Published:** 2023-10-12

**Authors:** Adam T. Evans, Heidi Vanden Brink, Jessica S. Lim, Brittany Y. Jarrett, Annie W. Lin, Marla E. Lujan, Kathleen Hoeger

**Affiliations:** 1 Department of Obstetrics and Gynecology, University of Rochester Medical Center, Rochester, NY 14620, USA; adam_evans@urmc.rochester.edu; 2 Department of Nutrition, Texas A&M University, College Station, TX 77843, USA; 3 Division of Nutritional Sciences, Cornell University, Ithaca, NY 14853, USA; 4 Hormel Institute, University of Minnesota, Austin, MN 55912, USA

**Keywords:** polycystic ovary syndrome, melatonin, sleep, sleep quality, ovary, circadian rhythm

## Abstract

Circulating melatonin is elevated in women with polycystic ovary syndrome (PCOS); whether circadian disruptions coincide with sleep disturbances in women with PCOS or their symptom severity is unclear. The objective of this observational pilot study was to determine whether altered patterns of melatonin excretion are associated with reduced sleep quality in women with versus without PCOS. Participants underwent a clinical assessment, transvaginal ultrasound, and reproductive hormone testing. Morning and evening urine samples were assayed for urinary 6-sulfatoxymelatonin (MEL) as a proxy for melatonin production. The night (morning MEL)-to-day (evening MEL) ratio, or N:D ratio, was determined to approximate the rhythm of MEL production. Sleep quality and duration were assessed using the Pittsburgh Sleep Quality Index (PSQI) and wrist actigraphy. No differences were detected in overnight MEL, daytime MEL, or the N:D ratio in participants with PCOS versus controls. The PCOS group experienced reduced weekend sleep efficiency vs. controls (81% vs. 88% *p* < 0.05). The number of follicles per ovary (FNPO) was positively associated with overnight MEL (r = 0.359, *p* < 0.05). Weekend sleep time and overnight MEL concentrations were dependent on PCOS status. Therefore, diagnostic features of PCOS were associated with MEL production and sleep disturbances, suggesting that women with a more severe clinical presentation of PCOS may be more likely to experience altered MEL production or sleep disturbances.

## 1. Introduction

Polycystic ovary syndrome (PCOS) is a reproductive disorder characterized by oligo-anovulation, hyperandrogenism, and polycystic ovarian morphology that affects approximately 10% of reproductive-aged females [1]. PCOS is associated with significant metabolic and psychological sequelae [2]—both of which are factors that have been linked to impaired sleep [3,4]. Females with PCOS are more likely to experience sleep disorders such as obstructive sleep apnea, as well as abnormalities in sleep architecture (i.e., a reduction in the percentage of rapid eye movement sleep) when compared to those without PCOS [5]. Prior studies have also shown that those with PCOS experience lower sleep quality, reduced sleep efficiency, and greater daytime sleepiness relative to healthy controls [5,6,7,8,9,10]. In addition to negatively impacting quality of life, impaired sleep may have far-reaching health implications for those living with PCOS, as aberrant sleep patterns have been associated with a more severe metabolic profile, including obesity and insulin resistance [6]. Therefore, the relevance of sleep in the pathophysiology of PCOS and its associated comorbidities is of growing interest.

Melatonin is synthesized and secreted by the pineal gland and predominantly functions as a chronobiotic to synchronize and facilitate entrainment of behavioral and physiological rhythms [11,12]. Melatonin is primarily excreted in urine as 6-sulfatoxymelatonin (MEL), which closely reflects plasma concentrations [13]. Melatonin is also hypothesized to exhibit endocrinologic effects beyond its circadian role, with recent evidence demonstrating antioxidant activity within the ovary [5]. Specifically, melatonin has been implicated in the regulation of ovarian sex hormone production and in the reduction in local reactive oxygen species (ROS), thereby preventing follicular atresia [14]. Therefore, it is plausible that disruptions in melatonin secretion may impact not only circadian rhythms but also reproductive function. Indeed, there is evidence for disrupted melatonin secretion among women with reproductive dysfunction, including those with PCOS. Namely, elevated 24 h total [13], morning [6,15], and nighttime [8] melatonin concentrations have been documented in women with PCOS. Further, women with PCOS were shown to have a reduced night-to-day (N:D) ratio of MEL, driven by increased daytime MEL concentrations [16]. Given that the N:D ratio can serve as a proxy for the circadian rhythm of MEL production, a lower N:D ratio or dampened magnitude of the night-to-day shift of MEL implies that the physiological control of melatonin secretion by the circadian timing system may be altered in the context of PCOS. Factors impacting altered melatonin dynamics in PCOS are unclear, as are their alignment with the degree of disordered sleep common in this condition. The role of androgens is inconsistent, as both positive [6] and negative [13] associations between melatonin and hyperandrogenism have been reported. Evidence linking the degree of menstrual cycle dysfunction and/or ovarian dysmorphology to altered melatonin secretion is also largely unexplored. Interestingly, analysis of ovarian granulosa cells obtained from infertile women with PCOS has demonstrated blunting of the rhythmic expression of some circadian clock genes, highlighting the critical impact such disruptions may have on gonadal function [17]. Ultimately, more data are needed to corroborate that disrupted circadian rhythms may occur concurrently with and/or contribute to the pathophysiology of PCOS while simultaneously contributing to the appearance of marked sleep disturbances in these women.

To address this knowledge gap, the primary objective of this observational pilot study was to determine whether altered patterns of day and night MEL excretion coincided with reduced sleep quality among women with PCOS. A secondary objective was to determine whether disrupted MEL production was associated with the diagnostic features of PCOS.

## 2. Materials and Methods

### 2.1. Study Subjects

In this ancillary study, participants were prospectively recruited at the University of Rochester or Cornell University (ClinicalTrials.gov NCT01859663, NCT01927432, NCT01927471, NCT01785719) from 2015–2019. Existing study protocols already collecting relevant reproductive and metabolic endpoints were amended to include sleep and circadian endpoints uniquely for this study. Participants were phenotyped according to criteria supported by the 2018 International Guideline for PCOS [18]. Specifically, participants with PCOS met at least two out of the three criteria: menstrual irregularity, biochemical or clinical hyperandrogenism (HA), and/or polycystic ovarian morphology on ultrasound (PCOM). Menstrual irregularity was defined as <21 or >35 days between menses based on self-reporting during hormone-free intervals. Biochemical HA was defined as either Free Testosterone (T) > 0.815 ng/dL, bioavailable T > 19.06 ng/dL, or free androgen index (FAI) >6%; clinical HA was defined using a modified Ferriman Gallwey (mFG) score of ≥6. PCOM was defined as at least one ovary with an ovarian volume (OV) > 10mL or >9 follicles in a single cross-section of the ovary (FNPS)—the latter of which has been shown to have high diagnostic accuracy for PCOS on ultrasound [19]. Exclusionary criteria included the use of medications known or suspected to interfere with reproductive function within 2 months of enrollment, melatonin supplementation, elevated prolactin (PRL), thyroid stimulating hormone (TSH), or follicle-stimulating hormone (FSH), current pregnancy or active breastfeeding. The non-PCOS reference group exhibited no more than one of the diagnostic criteria and is herein referred to as the control group.

### 2.2. Ethical Considerations

Written informed consent was obtained from the participants before any procedures were conducted. All parent protocols and study-specific amendments were approved by the Institutional Research Boards at Cornell University and the University of Rochester. Study procedures occurred at the Human Metabolic Research Unit (Cornell University, Ithaca, NY, USA) or Strong Fertility and Clinical Research Center (University of Rochester, Rochester, NY, USA).

### 2.3. Procedures

Participants attended a clinical research unit after an overnight fast for the following clinical assessments and procedures: (a) transvaginal ultrasound of the ovaries (b) reproductive and medical health history, (c) 75 g oral glucose tolerance test (OGTT), (d) physical exam and participant self-report to grade terminal hair growth using the modified Ferriman-Gallwey scoring system and (e) vitals and anthropometry assessment (waist and hip circumference, height, weight, blood pressure). Blood glucose was measured on-site using a glucometer (Accu-chek Aviva, Roche, Basel, Switzerland), and values were used to calculate the homeostatic model assessment for insulin resistance (HOMA-IR; (Insulin_0hr_ * Glucose_0hr_)/22.5) [20,21].

Participants with self-reported regular menstrual cycles were evaluated during the early follicular phase. Participants with self-reported irregular menstrual cycles were evaluated at random times. Participants were asked to reschedule their clinical assessments if investigators (1) observed the presence of a large follicle (>10 mm) or (2) had evidence of recent ovulation on the day of the ultrasound. Voluson ultrasound systems (GE, Milwaukee, WI, USA) using either a RIC5-9W-RS, RIC5-9A-RS, or RIC6-12-D endovaginal transducer were used to capture volumes of the ovaries. Images of the right and left ovaries were analyzed in 2D offline using a grid overlay [22] by trained members of the research team who achieved excellent agreement [intraclass correlation coefficient >0.9]. Follicle number per ovary (FNPO) represented the mean number of antral follicles 2-9 mm across both ovaries. Ovarian volume was determined using the following formula: [π * (average of all four linear measurements in orthogonal planes)] and reported as the mean of both ovaries, based on an internal study comparing different formulae to calculate ovarian volume in 2D versus 3D measures. If a dominant follicle was identified in one ovary, ovarian data from the other ovary were used in the event a follow-up ultrasound could not be scheduled. When poor image quality prevented reliable assessments in one of two ovaries, only the values of the ovary that could be visualized were analyzed by investigators.

### 2.4. Sleep-Related Endpoints

Sleep quality was assessed using the Pittsburgh Sleep Quality Index (PSQI), which is a valid and reliable 10-item questionnaire that reflects sleep habits for the most recent month based on self-report [23]. A score of less than or equal to 5 on the PSQI is associated with good sleep quality, whereas a score above 5 is associated with poor sleep quality.

Sleep measures were evaluated using a wrist-worn triaxial accelerometer. Participants were instructed to wear a triaxial accelerometer around their wrists at night and remove the device when out of bed for six consecutive nights. Participants recorded the dates and times the accelerometer was put on and taken off. The Sadeh Sleep Model [24] was used to compute overall, weekday night, and weekend night total sleep time and sleep efficiency. Sleep efficiency was reported as the percent of the time spent asleep divided by the time spent in bed. Participants were also instructed to obtain urine samples at home, collecting their first-morning void and evening (18:00–21:00) urine for two consecutive mornings and evenings during the week of wrist accelerometer wear. Participants were asked to record the dates and times of urine collection. Urine samples were stored in participants’ home freezers and were brought into their local clinical research unit on the ice. Urine samples were brought to room temperature for aliquoting and stored at −80 °C until analysis.

### 2.5. Assays

Urinary 6-sulfatoxymelatonin (MEL) was measured using the Buhlmann ELISA kit in triplicate (inter- and intra-assay variability <10%). Urinary MEL concentrations were adjusted for urinary creatinine. The average of the replicates was computed for analysis. The N:D ratio was computed as mean overnight MEL divided by mean daytime MEL for each of the two 24 h periods of data collection, totaling two N:D ratios. Follicle-stimulating hormone (FSH), luteinizing hormone (LH), estradiol (E2), sex hormone binding globulin (SHBG), prolactin (PRL), and thyroid stimulating hormone (TSH) were measured from serum using chemiluminescence immunoassay, Siemens Medical Solutions Diagnostics, Deerfield, IL (inter- and intra-assay variability <10%). Total testosterone was measured using LC/MS/MS by a laboratory associated with the Centers for Disease Control and Prevention’s Hormone Standardization Project (CDC-HoST) (Brigham Research Assay Core, Boston, MA, USA). FAI was calculated as Total T/SHBG (%). Free T and bioavailable testosterone were computed as described previously [25].

### 2.6. Statistical Analyses

Data were evaluated for normality, and the log was transformed as needed to meet statistical assumptions. Group comparisons were conducted using independent *t*-tests for continuous variables and Fisher’s exact tests for categorical variables. Associations between the diagnostic features of PCOS and sleep-related endpoints were evaluated using Pearson partial correlations after adjusting for BMI (Table A1). To test whether associations between sleep quality (i.e., PSQI) or accelerometer-defined sleep measures and MEL concentrations (day, night, or night-to-day ratio) differed between PCOS and control groups, multiple linear regression analyses were conducted. The dependent variables were MEL or N:D ratio, and the predictors included a sleep measure-by-PCOS interaction term. Data are presented as mean ± standard deviation (SD). Analyses were conducted using JMP Pro 14 (Cary, NC, USA). Statistical significance was set at *p* < 0.05. If participants had incomplete data collection for certain variables, they were still utilized in the analyses for which they had data. The original observational pilot sample size was determined via a power calculation to detect a difference in the mean N:D MEL ratio, which showed that six participants per group gave a statistical power of greater than 95% [16]. This sample size was preliminary, assuming a three-fold difference across groups, and was intended to generate effect sizes to inform a larger study.

## 3. Results

A total of forty-two participants were enrolled, with seven excluded due to oral contraceptive use (N = 6) and melatonin supplementation (N = 1). Twenty-two individuals met the criteria for PCOS, and thirteen met the criteria for controls. The demographic and clinical features of each group are shown in Table 1. There were no differences between groups with respect to BMI or age. By design and compared to controls, women with PCOS had significantly longer mean menstrual cycle lengths (*p* < 0.05), larger ovaries (*p* < 0.0001), increased follicle counts (FNPO, *p* = 0.0016, FNPS, *p* = 0.003), and were hyperandrogenic, as defined by hirsutism (*p* = 0.033) and TT (*p* = 0.03). Furthermore, 2 h insulin following an OGTT was higher (*p* = 0.01), and 2 h glucose also tended to be greater (*p* = 0.051) in PCOS versus controls.

**Figure 1:**
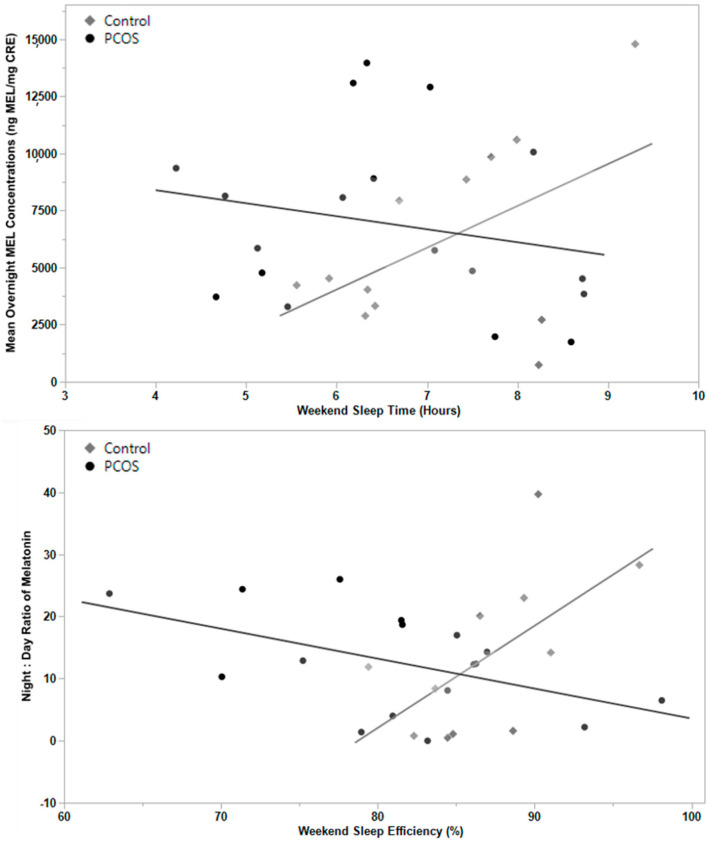
Associations between melatonin concentrations and weekend sleep quality are dependent on PCOS status. Associations between overnight melatonin production estimated using morning urinary MEL concentration and increasing weekend sleep time differ based on PCOS status (top panel; *p* < 0.05). Similarly, the association between N:D MEL ratios and weekend sleep efficiency differs based on PCOS status (bottom panel; *p* < 0.05).

Differences in sleep-related endpoints between groups are presented in Table 2. No differences in MEL-related or PSQI-defined sleep variables were detected between PCOS and controls (*p* > 0.05). With respect to the accelerometer-defined sleep variables, women with PCOS had significantly reduced weekend sleep efficiency when compared to controls (81% vs. 88%, *p* = 0.01). Regression analyses with a sleep measure-by-PCOS interaction term revealed that the association between overnight MEL concentration and accelerometer-defined sleep time on the weekend differed between those with and without PCOS (Figure 1; *p*_interaction_ = 0.0416). Similarly, the association between N:D MEL ratio and weekend sleep efficiency varied by PCOS status (*p*_interaction_ = 0.0017, N:D MEL (1); *p*_interaction_ = 0.0028, N:D MEL (2)). There were no statistically significant interactions between PCOS status and PSQI or other accelerometer-defined sleep measures on any MEL outcomes (*p* > 0.05). With respect to data collection, 5 participants had incomplete MEL data sets, 1 participant did not complete the PSQI, and four did not have accelerometer-defined sleep data.

Partial correlations were performed to assess the independent association between the diagnostic features of PCOS and urinary MEL endpoints, adjusting for BMI, which may have confounded the associations (Table A1). FNPO was positively associated with mean overnight MEL concentrations (*p* < 0.05; Table 3). No other significant associations between menstrual cyclicity, biochemical hyperandrogenism, or ovarian morphology and MEL endpoints were observed (*p* > 0.05).

## 4. Discussion

In this observational pilot study, we examined sleep quality in women with and without PCOS and tested whether an altered circadian rhythm output (i.e., urinary melatonin) is uniquely associated with poor sleep quality in individuals with PCOS. We also determined whether the degree of urinary melatonin excretion was associated with the severity of the diagnostic features of PCOS. Our data demonstrate that weekend sleep efficiency was lower in women with PCOS and that the association between weekend sleep time and overnight MEL concentrations is dependent on PCOS status. Furthermore, we report that FNPO is positively associated with mean overnight MEL concentration, which, to our knowledge, has not been previously reported.

Among all participants, mean overnight MEL was positively associated with FNPO but not hyperandrogenism, which was somewhat unexpected. The precise relationship between MEL and hyperandrogenism remains controversial, with previous studies reporting positive [6,26] or negative [13] associations between circulating androgens and MEL in PCOS. The relationship between MEL and ovarian morphology is also poorly established. We note two possible explanations for the associations between FNPO and MEL. First, it is plausible that in the present study, the degree of follicle excess (FNPO) may serve as a proxy for the phenotypic severity of PCOS [27] and that the severity of PCOS is associated with the magnitude of circadian rhythm disruption [15]. The severity of a PCOS phenotype may be related to the degree of circadian disruption as prior studies have demonstrated associations between melatonin and increased cardiovascular risk [16] and the degree of hyperandrogenism [6]—albeit not consistently. Second, increased MEL excretion may reflect ovarian inflammation and not hyperandrogenism. PCOS is characterized by a state of high oxidative stress [8], and melatonin has an antioxidant role as a free radical scavenger within the ovary [14]. Therefore, higher serum melatonin concentrations reported in PCOS may be a consequence of reduced intra-follicular melatonin concentrations via a negative feedback mechanism [14]. Furthermore, low intrafollicular melatonin has been associated with chronic anovulation and poor oocyte quality secondary to oxidative stress and a pro-inflammatory state [14]. A prior study examining human ovarian follicular fluid following in vitro fertilization cycles found that women with PCOS have lower intrafollicular melatonin levels than controls [28]. We posit that an increased FNPO in these patients may be indicative of pronounced ovarian dysfunction secondary to greater ovarian oxidative stress from a lower intrafollicular concentration of melatonin. The combination of these features, therefore, may contribute to aberrant folliculogenesis. However, the degree to which MEL (the urinary metabolite of melatonin) reflects the intra-ovarian melatonin milieu is not well established and was not measured in this study; therefore, additional research is needed to corroborate this hypothesis. To contextualize this argument, PCOS has been described as a pro-inflammatory state, with prior studies demonstrating elevated C-reactive protein levels in addition to markers of oxidative stress [29]. Furthermore, a study examining the impact of 12 weeks of oral supplementation with melatonin in patients with PCOS demonstrated a reduction in serum inflammatory markers [29]. With respect to possible clinical implications of these findings, a future exploration of whether oral melatonin supplementation impacts intrafollicular melatonin levels and whether this correlates with a decrease in FNPO and/or an improvement in menstrual cyclicity would allow elucidation of a possible therapeutic pathway.

We also observed a misalignment between overnight MEL and sleep efficiency in participants with PCOS. In women without PCOS, overnight MEL increased as anticipated alongside increased sleep duration relative to the time spent in bed (sleep efficiency). However, in participants with PCOS, as sleep efficiency increased, the magnitude of the overnight MEL rise relative to the daytime was significantly blunted. This observation supports previous reports of circadian misalignment in this population. An increased MEL [13,26], increased morning MEL [15], and a delayed MEL offset after wakening [6] have all been observed in women and adolescents with PCOS. We should note that not all studies report concomitant sleep disruptions in women with PCOS, which we posit may be attributed to heterogeneity in how circadian rhythms and sleep patterns are assessed across studies. With that said, our study contributes to a growing body of evidence that sleep quality, assessed quantitatively, and MEL may be misaligned in women with PCOS. Whether disrupted melatonin production and MEL excretion are a cause or a consequence of poor sleep quality in PCOS (in our case, reduced sleep efficiency) remains unclear and warrants further investigation.

In a departure from previous studies, we did not detect group differences in PSQI-defined measures of sleep quality [8]. Similarly, we did not detect differences between PCOS and non-PCOS groups with respect to measures of circadian rhythm (as represented by overnight MEL, daytime MEL, or the N:D ratio), which contrasts previous studies reporting such findings [6,16]. However, our finding that weekend sleep efficiency is significantly reduced in the PCOS group is consistent with other studies [6,8,30]. Our null findings were unexpected, as co-morbid sleep disorders are known to be common in women with PCOS [7,9]. Likewise, insulin resistance is associated with both obstructive sleep apnea [7,31] and PCOS. Indeed, women in the PCOS group had higher insulin levels following an OGTT. However, as a group, they did not exhibit worse sleep quality. It is plausible that our participant pool—both the PCOS and control groups—may have blunted any effects of PCOS status. On average, neither group met the nightly sleep recommendation defined by the American Academy of Sleep Medicine (7–9 h per night) during the period of data collection, which is likely to have diluted any possible effect attributed to PCOS status [32]. It is also plausible that our definition of controls may have reduced the effect size attributed to PCOS status. Controls were defined as having no more than 1 of the diagnostic features of PCOS; therefore, they may have exhibited some mild evidence of reproductive dysfunction. As an observational pilot study, direct clinical correlations cannot be drawn from our results; however, our findings of a misalignment between MEL and sleep efficiency in participants with PCOS, as well as reduced weekend sleep efficiency, highlight the importance of discussing sleep and sleep hygiene during routine clinical evaluation. This is especially relevant, given the association between abnormal sleep, metabolic derangements, and insulin resistance—factors with well-established links to PCOS [6].

The study had several strengths. Participants were prospectively recruited and well-phenotyped by the research team using detailed reproductive health histories, standardized testosterone assays, and rigorous ovarian imaging. We conducted a comprehensive assessment of metrics to assess sleep quality and circadian rhythm, which enabled comprehensive assessments of qualitative and quantitative sleep quality alongside MEL. The study also had limitations. Although we provided instructions for week-long accelerometer wear, participant compliance represented a significant challenge in capturing sleep and circadian shifts, and therefore, wrist-worn accelerometer wear was varied (total number of weekday nights data obtained: 3 to 5; total number of weekend nights data obtained: 1 to 2). Moreover, because the timing of urine can greatly reflect the degree to which overnight MEL production is captured or missed [33], it is plausible that the null findings may be partially attributed to inconsistent timing of urine collection (first morning voids among participants ranged from 04:00–12:00). We acknowledge that measurement of urinary 6-sulfatoxymelatonin in urine using ELISA may significantly overestimate circulating MEL levels [34] and that our approach abrogates any definitive conclusions regarding the exact timing and amplitude of MEL secretion. Future studies would benefit from the assessment of dim light onset MEL (DLMO) in plasma or salivary samples collected every 30 min to 1 h during a constant dim light protocol and assayed using mass spectrophotometry. Reduced compliance also resulted in missing values, which may further reduce the ability to detect differences across groups. The nature of this observational pilot study did not allow for subdivision by PCOS phenotype; therefore, the impact of phenotypic severity per se, on circadian disruption could not be elucidated. With respect to sample size, our analysis determined that we were sufficiently powered to detect a statistically significant difference when investigating the mean N:D MEL ratio as an outcome. However, future research should include larger sample sizes to establish clinically meaningful differences (i.e., threshold of clinical significance) to investigate among the different PCOS phenotypes. Finally, one participant reported a history of obstructive sleep apnea during study participation, which may lower sleep quality due to nighttime awakening. Although it is unlikely that one participant could drive an effect size, future studies should carefully screen participants for sleep-disordered breathing at the time of enrollment, given the increased risk of OSA in women with PCOS, even after controlling for BMI [5].

## 5. Conclusions

In summary, we report reduced weekend sleep efficiency and evidence of misalignment between circadian rhythms and sleep quality in participants with PCOS. We also report that the degree of overnight MEL is positively associated with increasing follicle number, suggesting that the severity of a PCOS phenotype may be linked to circadian disruption. Due to the heterogeneity in sample collection, additional research is needed to corroborate these findings. However, these data provide formative evidence that the pathophysiology of PCOS may be closely related to circadian disruption, and there is fertile ground for further clinical investigation.

## Figures and Tables

**Table 1:** Demographic and clinical features.

	Control		PCOS		
	Mean	Std Dev	Mean	Std Dev	*p* Value
N	13		22		
Age (y)	28.0	5.8	28.3	5.5	NS
Age at menarche (y)	12.2	0.9	13.1	1.1	0.018
** Anthropometry and glucoregulation **					
BMI (kg/m^2^)	31.3	4.49	30.9	9.41	NS
WHR	0.83	0.08	0.82	0.08	NS
Body fat (%)	39.4	5.43	39.7	10.21	NS
Insulin (0 HR, mIU/mL)	7.6	2.77	13.9	12.47	NS
Glucose (0 HR, mg/dL)	90.5	5.83	93.3	11.96	NS
HOMA-IR	1.7	0.63	3.5	3.79	NS
Insulin (2 HR, mIU/mL)	28.5	12.70	73.2	64.67	0.010
Glucose (2 HR, mg/dL)	81.8	87.20	101.6	38.18	0.051
Systolic BP (mmHg)	117	17.6	115	15.6	NS
Diastolic BP (mmHg)	72	19.4	69	9.7	NS
** Diagnostic features of PCOS **					
MCL (days)	30	2.2	67	72.3	0.001
Hirsutism score	3	2.9	8	6.2	0.005
TT (ng/dL)	18.7	14.01	36.7	26.36	0.033
FAI (%)	1	1.0	4	3.6	0.071
FT (ng/dL)	0.25	0.18	0.58	0.52	0.075
BIOT (ng/dL)	5.9	4.42	13.6	12.11	0.072
Mean OV (cm^3^)	5.4	1.31	9.7	2.90	<0.001
Mean FNPO	19	10	40	20	0.002
Mean FNPS	6	3	11	4	<0.001
** Reproductive endocrinology **					
LH (mIU/mL)	4.3	1.96	8.49	5.83	0.009
FSH (mIU/mL)	6.6	2.62	5.28	1.79	NS
SHBG (nmol/L)	54.6	31.90	57.77	35.65	NS

*p*-values reflect two-sided *t*-tests. Variables were transformed as needed (logarithmic or square root) to meet assumptions. Ovarian analyses reflect mean values, except when a dominant follicle (DF) or corpus luteum (CL) was present, then the ovarian data reflect the ovary that did not contain the DF or CL. Abbreviations: BMI, body mass index; WHR, waist-hip ratio; HOMA-IR, homeostasis model assessment for insulin resistance; MCL, mean cycle length; TT, total testosterone; FAI, free androgen index; FT, free testosterone; BIOT, bioavailable testosterone; Mean OV, mean ovarian volume; Mean FNPO, mean follicle number per ovary; Mean FNPS, mean follicle number per single cross-section; LH, luteinizing hormone; FSH, follicle stimulating hormone; SHBG, sex hormone binding globulin; Fasting glucose and insulin levels (0HR) prior to administration of a 75 g oral glucose tolerance test (OGTT) and 2 h afterward (2HR).

**Table 2:** Differences in sleep-related endpoints between PCOS and controls.

Urinary Melatonin Variables	Controls		PCOS		
	Mean	Std Dev	Mean	Std Dev	*p* Value
Mean overnight MEL (ng MEL/mg CRE)	6213.07	4146.26	6972.42	3584.04	NS
Mean daytime MEL (ng MEL/mg CRE)	865.93	643.20	1297.28	1122.41	NS
Night/day ratio (1)	15.76	16.06	13.41	8.72	NS
Night/day ratio (2)	13.60	13.01	11.49	8.57	NS
** PSQI-defined sleep variables **					
PSQI total	5	2.7	7	4.0	NS
Good sleep quality, N (%)	9 (69)		12 (55)		NS
Poor sleep quality, N (%)	4 (31)		10 (45)		
** Accelerometer-defined sleep variables **					
Total sleep time (h/day)	6.5	0.80	6.5	0.94	NS
Total weekday sleep time (h/day)	6.3	1.28	6.5	1.05	NS
Total weekend sleep time (h/day)	7.1	1.16	6.5	1.46	NS
Total sleep efficiency (%)	84	6.9	82	7.7	NS
Weekday sleep efficiency (%)	82	10.1	81	7.6	NS
Weekend sleep efficiency (%)	88	5.0	81	8.3	0.010

Variables were transformed as needed (logarithmic or square root) to meet assumptions. Means and SD reflect raw data. Implausible values (sleep efficiency > 100%) were excluded from their endpoint-specific analyses. Categorical analyses were conducted using Fisher’s Exact tests (two-sided). A PSQI score of >5 is categorized as poor sleep quality. MEL, 6-sulfatoxymelatonin; CRE, serum creatinine; PSQI, Pittsburgh Sleep Quality Index.

**Table 3:** Associations between diagnostic features of PCOS and urinary melatonin.

	MCL	TT	FT	FAI	Bio T	OV	FNPS	FNPO
Mean overnight MEL (ng MEL/mg CRE)	−0.016	0.082	0.131	0.137	0.127	0.120	0.298	0.359 *
Mean daytime MEL (ng MEL/mg CRE)	0.177	0.074	0.093	0.087	0.092	0.215	0.137	0.206
N:D ratio (1)	0.134	0.136	0.208	0.218	0.204	0.123	0.299	0.246
N:D ratio (2)	−0.215	−0.001	−0.069	−0.076	−0.070	−0.114	0.047	0.074

Partial correlations between urinary melatonin concentrations and diagnostic features of PCOS. Partial correlations were adjusted for any potential confounding effects of BMI (Table A1). In the cases of women with dominant follicles, ovarian data from the other ovary were reported (N = 6) or excluded if in the luteal phase (N = 1). In this case, ovarian endpoints from the other ovary are presented. One participant was excluded from all ovarian analyses because both ovaries had DFs. Abbreviations: MEL, melatonin; CRE, creatinine; MCL, mean menstrual cycle length; TT, total testosterone; FT, free testosterone; FAI, free androgen index; Bio T, bioavailable testosterone; OV, ovarian volume; FNPS, follicle number per single cross-section; FNPO, follicle number per ovary; CRE, serum creatinine. *, *p* < 0.05.

## Data Availability

The data presented in this study are available upon reasonable request from the corresponding author.

## Appendix A

**Table A1:** Parametric Associations Between Potential Metabolic Confounders and Covariates.

	BMI (kg/m^2^)	Fasting Insulin	2HR Insulin	HOMA-IR	% Fat
Overnight MEL (ng MEL/mg CRE)	−0.387 *	−0.306	−0.304	−0.298	−0.531 ***
Daytime MEL (ng MEL/mg CRE)	0.087	0.137	0.309	0.172	0.164
Night/Day Ratio (1)	−0.097	−0.003	−0.060	−0.038	−0.220
Night/Day Ratio (2)	−0.262	−0.248	−0.367 *	−0.269	−0.346 *
MCL (days)	0.285	0.376 *	0.391 *	0.409 *	0.260
TT (ng/dL)	0.092	0.338 *	0.364 *	0.312	0.049
FT (ng/dL)	0.365 *	0.510 **	0.530 ***	0.498 **	0.258
FAI (%)	0.442 **	0.560 ***	0.567 ***	0.556 ***	0.313
Bio T (ng/dL)	0.364 *	0.508 **	0.529 ***	0.497 **	0.255
OV (mL)	0.077	0.119	0.367 *	0.118	0.033
FNPS	0.118	0.307	0.538 **	0.290	0.091
FNPO 2–9 mm	0.214	0.336	0.535 **	0.326	0.105

Variables were log-transformed as needed to meet assumptions. In the cases of women with DFs, ovarian data from the other ovary were reported (N = 6) or were excluded if in the luteal phase (N = 1). In this case, ovarian endpoints from the other ovary are presented. One participant was excluded from all ovarian analyses because both ovaries had DFs. Abbreviations: MEL, melatonin; MCL, mean menstrual cycle length; TT, total testosterone; FT, free testosterone; FAI, free androgen index; Bio T, bioavailable testosterone; OV, ovarian volume; FNPS, follicle number per cross-section; FNPO, follicle number per ovary. *, *p* < 0.05; **, *p* < 0.01; ***, *p* < 0.001.
